# Higher Muscle Volume is Inversely Related to Chronological and Brain Age While Increased Visceral to Muscle Fat Ratio is Positively Related to Chronological and Brain Age

**DOI:** 10.1002/alz70856_106692

**Published:** 2026-01-08

**Authors:** Cyrus A. Raji, Somayeh Meysami, Soojin Lee, Saurabh Garg, Nasrin Akbari, Rodrigo Solis Pompa, Ahmed Gouda, Thanh Duc Nguyen, Saqib Basar, Yosef Gavriel Chodakiewitz, David A. Merrill, Amar Patel, Daniel J. Durand, Sam Hashemi

**Affiliations:** ^1^ Mallinckrodt Institute of Radiology, Washington University in St. Louis, St. Louis, MO, USA; ^2^ Pacific Brain Health Center, Pacific Neuroscience Institute and Foundation, Santa Monica, CA, USA; ^3^ Saint John's Cancer Institute at Providence Saint John's Health Center, Santa Monica, CA, USA; ^4^ Vigilance Health Imaging Network Inc., Vancouver, BC, Canada; ^5^ Prenuvo, Palo Alto, CA, USA

## Abstract

**Background:**

Brain age predicted from structural brain images on T1 weighted scans can lend insight to Alzheimer's risk factors such as muscle loss with sarcopenia. We thus investigated the link between body MRI measured muscle mass, muscle to fat ratio and brain age.

**Method:**

In all, 1,164 healthy participants from four sites (mean chronological age 55.17 ± 12.37 years, 52% women; 48% men; 39% non‐white) were scanned on 1.5T MR machines with a whole‐body protocol. Whole body sequences utilized in the quantitative analyses of muscle mass were coronal T1 were used to segment total muscle volume normalized to participant height, visceral adipose tissue (VAT) and subcutaneous adipose tissue (VAT). In this process, a nnU‐Net model was used for fully supervised segmentation and ITK‐SNAP was used for manual annotation. Brain age was computed from T1 MPRAGE scans using a regression‐based 3D Simple Fully Convolutional Network. The model was trained on in‐house T1‐weighted MRI scans collected from 5,500 healthy individuals, aged 18 to 89 years. Brain age gap (BAG) was calculated by subtracting chronological age from brain age. Bivariate correlations between total normalized muscle volume (TNMV) as well as VAT and SAT normalized to total muscle volume to chronological and brain age were done with partial correlations adjusted for sex with brain age analyses.

**Result:**

Mean brain age was higher than chronological age (56.04 ± 12.65, mean BAG = 0.69). Higher TNMV was related to both decreased chronological age (rp=‐0.2579, *p* = 2.524e‐17) and brain age (rp =‐0.2497, *p* = 2.65e‐16). VAT normalized to total muscle volume was linked to higher chronological (rp=0.3755, *p* = 2.615e‐36) and brain age (rp=0.3797, *p* = 3.871e‐37) adjusting for sex. No statistically significant links were noted with TNMV, VAT, SAT or and BAG. SAT was also not correlated in a statistically significant way to chronological or brain age.

**Conclusion:**

Increasing muscle mass is related to lower chronological and brain age while visceral fat normalized to muscle volume is related to increased chronological and brain age. Lack of correlation to BAG may be due to the relatively low BAG in this sample. This work suggests improving muscle mass and reducing visceral fat may improve brain aging.

